# Red blood cell transfusions impact response rates to immunotherapy in patients with solid malignant tumors

**DOI:** 10.3389/fimmu.2022.976011

**Published:** 2022-09-08

**Authors:** Rebekka Mispelbaum, Sandra Tessa Hattenhauer, Peter Brossart, Annkristin Heine

**Affiliations:** Department of Oncology, Hematology, Rheumatology and Immune-Oncology, University Hospital Bonn, Bonn, Germany

**Keywords:** immunotherapy, response, red blood cell transfusion, tumor, immunosuppression

## Abstract

Red blood cell (RBC) transfusions have been shown to exert immunosuppressive effects in different diseases. In consequence, RBC transfusions might also negatively influence the response to immunotherapeutic treatment approaches. To address how RBC transfusions impact response rates of antitumor immunotherapy (IT), we conducted a retrolective clinical study of patients with different solid tumors treated with IT (atezolizumab, pembrolizumab, nivolumab and/or ipilimumab). We assessed the number of RBC concentrates received within 30 days before and 60 days after the start of IT. Primary objective was the initial therapy response at first staging, secondary objectives the number of immune related adverse events and infections. 15 of 55 included patients (27.3%) received RBC concentrates. The response rates were 77.5% in the non-transfused (n=40) versus 46.7% in the transfused patient group (n=15) and reached statistical significance (p=0.047). The correlation between therapy response and transfusion was statistically significant (p=0.026) after adjustment for the only identified confounder “line of therapy”. In contrast, transfusion in the interval 30 days before IT showed no significant difference for treatment response (p=0.705). Moreover, no correlation was detected between RBC transfusion and irAE rate (p=0.149) or infection rate (p=0.135). In conclusion, we show for the first time that the administration of RBC transfusions during, but not before initiation of IT treatment, negatively influences the response rates to IT. Our findings suggest a restrictive transfusion management in patients undergoing IT to receive optimal response rates.

## Introduction

In the last two decades, a better understanding of the interaction of immune and tumor cells has arisen. Malignant tumors suppress the immune responses *via* signaling pathways including programmed death-ligand 1 (PD-L1) and programmed cell death protein 1 (PD-1) (1). An inhibition of these molecules, exploiting immune checkpoint inhibitors (CPIs), leads to an enhancement of tumor-directed CD8^+^ T-cell activity and in part to a reduction of Treg-depended immunosuppression resulting in durable remissions in a subgroup of patients with different tumor types (1, 2). Despite a current increasing use of immunotherapy (IT) in cancer care, factors that affect the efficacy of IT are still not completely identified (3). Based on the known immunomodulation, any interference with the immune system is likely to impact the response to IT, as well as risk for immune-related adverse events (irAEs) and infections during IT, suggesting that the role of concomitant treatments with immune-modulatory potential is of essential importance (4, 5). For instance, baseline use of steroids in non-small cell lung cancer patients treated with CPIs was associated with a worse outcome (6).

Red blood cell (RBC) transfusions are commonly used during cancer therapy, due to tumor-associated anemia or treatment-related toxicity (7). A known side effect of allogenic RBC transfusion is a transfusion-related immunomodulation (TRIM) resulting in modulation of the immune system (8, 9). RBC concentrates consist of cellular components (erythrocytes, residual leukocytes) as well as of humoral components (cytokines, soluble HLA peptides, extracellular micro-vesicles). All these factors are discussed triggers for TRIM, which underlying mechanism is not completely understood. As part of the suspected pathomechanism an attenuation of T cell function as well as an increased activation of immunosuppressive regulatory T cells (TREGs) are assumed (10–12).

The potential immunosuppressive effect of RBC transfusions has gained an increased awareness in oncology. In cancer surgery, a raised risk of tumor relapse for RBC transfusion recipients is currently investigated (9). One hypothesis is a pro-tumor environment for residual tumor cells caused by TRIM (9). For instance, there is emerging data for the clinical significance in colorectal cancer surgery (13). Despite the essential role of potential immunomodulating factors for IT, RBC transfusion has not been investigated in IT patients in detail.

In summary, RBC transfusions may display a relevant immunosuppressive effect acting as an opponent to immunotherapy. The aim of this study was to approach the question whether RBC transfusions impact outcome of IT in cancer patients. Furthermore, we wanted to dissect the influence of RBC transfusions on the risk of irAEs and infections.

## Methods

We conducted a retrolective clinical study of patients treated with IT for metastatic cancer at a single tertiary care center between 05/2015 and 10/2021. Inclusion criteria were at least one radiological staging after start of IT, a minimum survival time of 60 days to ensure a sufficient observation period and at least one immune status assessed by flow cytometry.

IT could be applicated as monotherapy, IT doublet as well as concomitant to radiation or any systemic therapies, including chemotherapy and targeted therapies. Investigated drugs were the PD-L1 inhibitor atezolizumab, the PD-1 inhibitors nivolumab and pembrolizumab as well as the CTLA-4 inhibitor ipilimumab.

For each cancer patient, we assessed the number of packed RBC transfusions received within 30 days before start of IT and within the first 60 days after start of IT. Patients were allowed to receive platelet transfusions during investigation period. Intravenous immunoglobulins, cryoprecipitates or plasma were not transfused.

Primary objective was the initial therapy response. Therefore, we considered the first conducted CT scan or MRI after the start of IT, which were evaluated by our radiologists according to the local hospital guidelines. If this imaging was performed within the first 60 days only the given transfusions up to this date were taken into account (RBC transfusion had to be given at least one day before imaging). Therapy response was defined as stable disease, partial response or complete response. Secondary objectives were the number of irAEs in the first 60 days after start of IT and the number of infections within 30 days before start of IT. Only infections treated with antibiotics were considered.

Data was collected by discharge letters, documented radiological examination or any electronical medical record.

### Statistical analysis

Demographic and baseline clinical characteristics (at time of initiation of IT) were analyzed using descriptive statistics. All variables included in this analysis had ≤5% missing. Differences between these two groups were tested by Fisher’s exact test and Wilcoxon test. We separately compared transfused and non-transfused patient groups for the transfusion time frames 30 days before and 60 days after start of IT.

To evaluate the association between treatment response and transfusion, bivariate analyses by Fisher’s exact test and Wilcoxon test were applied. To measure the effect of the specific number of packed RBC transfusions (after start of IT) for treatment response Odds were applied.

A bivariate analysis by Fisher’s exact test was performed to define the relationship between therapy response (dependent variable) and influencing parameters (sex, age, ECOG, type of IT, concomitant therapy, number of metastatic sites, LDH). In case of a statistically and clinically significant relationship, the variables were included in a multivariable logistic regression. For each variable, we calculated Odds ratio (ORs).

To evaluate the association between transfusion and irAE rate, respectively infection rate, bivariate analyses by Wilcoxon test were conducted.

All analyses and figures were performed using STATA software (version 15.1) and GraphPad Prism 5. A p<0.05 was considered as statistically significant. The study was conducted in accordance with the Declaration of Helsinki and was approved by the ethics committee of the medical department of the university Bonn (#340/21). Only previously documented data were used, no informed consent was needed. All patients were anonymized through the use of codes.

### Data availability

The data generated in this study are not publicly available due to patient data privacy but are available upon reasonable request from the corresponding authors.

## Results

### Baseline characteristics

59 patients in total diagnosed with metastatic cancer treated with IT were analyzed. 4 patients were excluded for further analysis due to death within 60 days after start of IT. The final population consisted of 55 patients. The mean age was 62 years (range: 34-90). The gender distribution of the study population was 70.9% male and 29.1% female. ECOG performance status 0-1 was documented for 35 patients (63.6%). In 29 patients (52.8%) IT was administered as first or second line treatment. 27.3% of patients were treated in addition to IT with chemotherapy and 20.0% of patients with radiotherapy(**Table 1**). 15 of the analyzed patients (27.3%) received RBC transfusions while 40 patients (72.7%) had no indication for RBC transfusion in the first 60 days after start of IT. Both cohorts were equally distributed by age (p=0.059), sex (p=0.184), ECOG (p=1.000) and burden of disease (number of organs with metastasis p=0.912; LDH p=0.441). While tumor type was mostly balanced between the two groups, the proportion of lung cancer was higher in the non-transfused patient group. Both cohorts did not differ statistically significant with regard to concomitant therapy as chemotherapy (p=0.461) or radiation (p=1.000). Compared to non-transfused patients, transfused patients were more likely to get a two-drug IT (p=0.011). Transfused patients were also more likely to receive IT in a higher therapy line (p=0.016) (**Table 1**). The transfused patient group received on average 4 RBC concentrates per person (median: 2 RBC). The baseline characteristics of patients transfused during 30 days before therapy start are described in the supplements (**Supplementary Table 1**).

**Table 1:** Patient characteristics of transfused and non-transfused patients (in the first 60 days after IT start).

	total(N = 55), n (%)	RBC transfusion (N = 15), n (%)	no RBC transfusion (N = 40), n (%)	p-value^a^
**Age (years)**				
Mean	62	56	65	0.059*
Range	34-90	34-82	37-90	
Elderly (>70)	15 (27.3)	2 (13.3)	13 (32.5)	0.192
**Gender**				0.184
Male	39 (70.9)	13 (86.7)	26 (65.0)	
Female	16 (29.1)	2 (13.3)	14 (35.0)	
**ECOG**				1.000
0-1	35 (63.6)	9 (60.0)	26 (65.0)	
≥2	17 (30.9)	5 (33.3)	12 (30.0)	
**Primarius**				0.414
Lung	19 (34.5)	2 (13.3)	17 (42.5)	
Skin	7 (12.7)	2 (13.3)	5 (12.5)	
Head neck	10 (18.2)	4 (26.7)	6 (15.0)	
Urogenital	5 (9.1)	2 (13.3)	3 (7.5)	
CUP	4 (7.3)	1 (6.7)	3 (7.5)	
Breast	3 (5.5)	1 (6.7)	2 (5.0)	
Others	7 (12.7)	3 (20.0)	4 (10.0)	
**One drug Immunotherapy**				0.011
Atezolizumab	8 (14.5)	2 (13.3)	6 (15.0)	
Pembrolizumab/Nivolumab	34 (61.8)	6 (40.0)	28 (70.0)	
Ipilimumab	1 (1.8)	0	1 (2.5)	
**Two drug immunotherapy**				
Nivolumab+Ipilimumab	12 (21.8)	7 (46.7)	5 (12.5)	
Additional chemotherapy	15 (27.3)	3 (20.0)	12 (30.0)	0.461
Additional radiotheraphy	11 (20.0)	3 (20.0)	8 (20.0)	1.000
**Line of therapy**				0.016
1	14 (25.5)	0	14 (35.0)	
2	15 (27.3)	4 (26.7)	11 (27.5)	
3	13 (23.6)	7 (46.7)	6 (15.0)	
4	5 (9.1)	2 (13.3)	3 (7.5)	
≥5	8 (14.5)	2 (13.3)	6 (15.0)	
Mean	2.7	3.4	2.5	
**Number of organs with metastasis**				0.912*
Mean	1.7	1.7	1.8	
LDH				0.441*
Mean	317	457	262	

(a) was calculated using Fisher's exact/ (*) Wilcoxon. RBC, red blood cell; CUP, cancer of unkown primary.

### Patients without concomitant RBC transfusions within 60 days after IT initiation show better outcome than those who receive RBC transfusions

**Figure 1:**
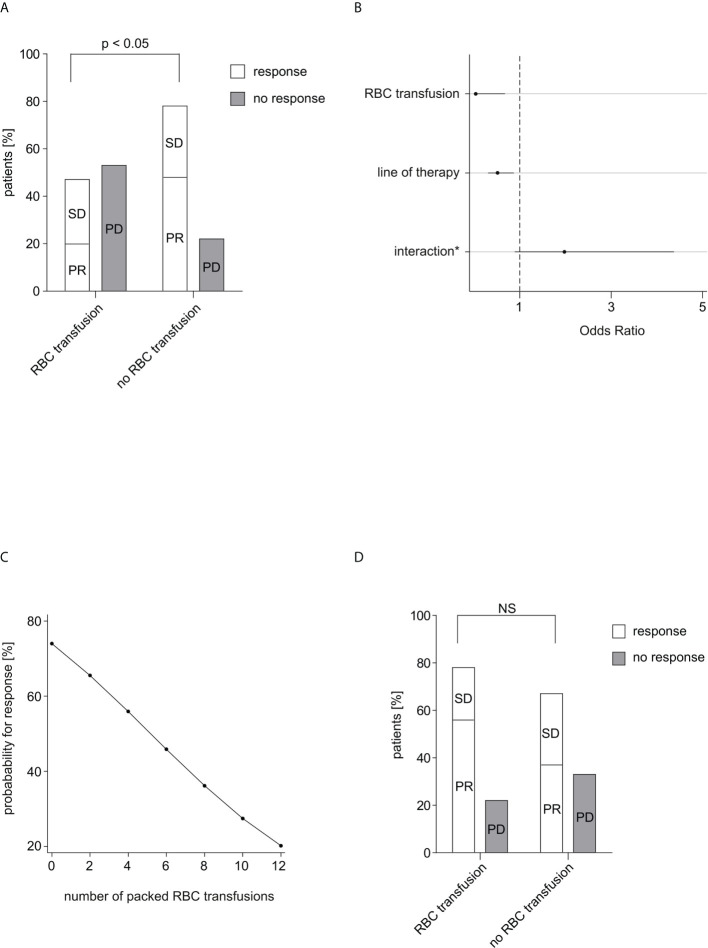
Response rate in relation to RBC transfusions. **(A)** The response rate depending on received RBC transfusions within 60 days after start of IT is shown. Complete response, partial response and stable disease were considered as response. Of 40 patients in the non-transfused group 31 patients (77.5%) responded to IT, while of 15 patients in the transfused group only 7 patients (46.7%) responded to IT. The difference was calculated using Fisher’s exact test (p=0.047). **(B)** Multiple logistic regression analysis of therapy response to IT. (*) between RBC transfusion and line of therapy. After adjustment for the effect of the confounding factor “line of therapy” the correlation between therapy response and transfusion was statistically significant (p=0.026). No other confounding factors could be identified. **(C)** The probability for response in dependence on the number of transfused packed RBC units within 60 days after start of IT is depicted. **(D)** The response rate depending on received RBC transfusions within 30 days before start of IT is shown. Of 46 patients in the non-transfused group 31 patients (67.4%) responded to IT, while of 9 patients in the transfused group 7 patients (77.8%) responded to IT. The difference was calculated using Fisher’s exact test (p=0.705). RBC, red blood cell; IT, immunotherapy; PR, partial response; SD, stable disease; PD, progressive disease; NS, not significant.

The average time until first CT/MRI scan in our study was 69 days. Treatment response was detected in 38 of 55 patients, thereof 16 (29.1%) with stable disease, 22 (40.0%) with partial response. 17 patients (30.9%) showed no response to IT. Response rates were investigated depending on transfusion of RBC concentrates within 60 days after start of IT. In the non-transfused patient group (n=40), a response rate of 77.5% was found, whereas in the transfused patient group (n=15) only 46.7% responded to IT. The difference was statistically significant at Fisher’s exact test (p=0.047) (**Figure 1A**).

There was no relevant correlation between therapy response and respectively age (p=0.126), sex (p=0.533), ECOG (p=1.000), type of IT (one drug or IT doublet; p=0.158), concomitant therapy (radiotherapy/chemotherapy; p=0.471/0.109), number of organs with metastasis (p=0.802), nor LDH (p=0.724). In contrast, we detected a statistically significant difference in treatment response depending on the line of therapy (p=0.026). Since the response rate of IT declined with a higher therapy line, it was assessed as a clinical expected and principal confounder in the following analyses.

The correlation between therapy response and RBC transfusion was statistically significant (p=0.026) after adjustment for the effect of the confounding factor line of therapy in a multiple logistic regression. The interaction of transfusion and line of therapy showed no statistically significance (p=0.092). The effect of RBC transfusion on therapy response was substantially stronger than the effect of line of therapy (OR=0.037 vs. OR=0.515). The Odd for therapy response was 97% lower under any transfusion while the Odd for therapy response decreased around 49% per therapy line (**Figure 1B**).

The examination of the exact number of received packed RBC transfusions showed a non-statistically significant decrease about 18.3% in the Odd for treatment response per RBC concentrate (p=0.121). The probability for response decreased almost linear in dependence of number of transfused RBC units (**Figure 1C**).

### Patients receiving RBC transfusions within 30 days before IT initiation show similar outcomes than those without RBC transfusions

Within 30 days before IT, 9 patients (16.4%) received RBC transfusion, while 46 patients (83.6%) did not. 7 of the transfused patients (77.8%) and 31 of the non-transfused patients (67.4%) responded to IT. In contrast to concomitant IT-RBC transfusions, patients with or without transfusion in the interval 30 days before IT showed no significant difference for treatment response (p=0.705) (**Figure 1D**).

### RBC transfusions do not impact on the rate of irAEs nor infections

**Figure 2:**
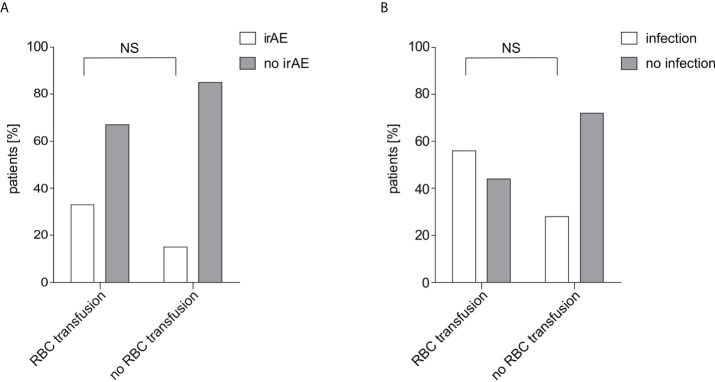
IrAE and infection rate in relation to RBC transfusions. **(A)** The irAE rate depending on received RBC transfusions within 60 days after start of IT is shown. Of 40 patients in the non-transfused group 6 patients (15.0%) showed an irAE while of 15 patients in the transfused group 5 patients (33.3%) showed an irAE. The difference was calculated using Fisher’s exact test (p=0.149). **(B)** The infection rate depending on received RBC transfusions within 30 days before start of IT is shown. Of 46 patients in the non-transfused group 13 patients (28.3%) were treated with antibiotic treatment, while of 9 patients in the transfused group 5 patients (55.6%). The difference was calculated using Fisher’s exact test (p=0.135). RBC, red blood cell; irAE, immune-related adverse events; NS, not significant.

No statistically significant relation was found between irAE rate and transfusion of RBC concentrates (p=0.149) nor infection rate and transfusion (p=0.135). In total, 11 patients (20.0%) showed an irAE within 60 days after start of IT. In the non-transfusion group the irAE rate was 15.0% and in the transfusion group 33.3% (**Figure 2A**). Antibiotic treatment within 30 days before start of IT was reported for 18 patients (32.7%). Infection rate was 28.3% in the non-transfusion group and 55.6% in the transfusion group (**Figure 2B**).

## Discussion

The use of IT in anti-cancer treatment approaches is rapidly increasing (14). Several studies have analyzed concurrent medications and their potential for damping the activity of immunotherapies (3, 4). An immunosuppressive effect is likewise assumed for RBC transfusion (8, 9). Due to the underlying disease and the hematotoxic effect of therapy, many cancer patients develop anemia that require transfusions (15). The potential immunosuppressive effects suggest that RBC concentrates might influence the response rate to IT. So far, the role of packed RBC transfusion for IT response has not been investigated in detail. The main objective of our study was to define the impact of RBC transfusions before and during IT on therapy outcome. Our analysis identified a negative influence of RBC transfusions, received during the first 60 days of IT, on the likelihood of responding to IT in patients with advanced malignancies. Our findings are supported by a large meta-analysis of Amato and Pescatori including 36 studies on 12,127 patients with a curative resection of colorectal cancer and a perioperative RBC transfusion. Between recurrence and RBC transfusion the estimated Odds ratio was 1.42 (95% CI, 1.20 to 1.67) (16).

There is an ongoing discussion about the relationship between a higher physiologic severity of illness and the need for supportive therapy (RBC transfusion, steroid administration), the latter being probably just a prognostic marker (17). To do this justice, we first compared our two cohorts of transfused and non-transfused patients. We could not detect any statistically significant differences for disease severity or performance status (number of organs with metastasis, LDH, ECOG, age). Secondly, we excluded patients who died within the first 60 days, thereby ensuring that patients with too advanced, aggressive disease with very low chance of therapeutic response were ruled out. Due to the retrolective design and the inhomogeneous small cohort, there remained at least a partly hindered comparability of the groups.

Our observational study is based on a detailed data collection considering different potential confounders. The statistical analysis showed a relevant influence of the line of IT on treatment response. Poorer treatment response and an increasing need for RBC transfusions in higher lines of therapy caused by accumulated therapy-associated bone marrow toxicity is expected (15). Therefore, therapy line had to be included as a confounder in our statistical model. After adjustment, there was an independent strong statistically significant effect of RBC transfusions on therapy response. Interestingly, we were able to show that an increasing number of transfused units were associated with a reduction of response rate. Amato and Pescatori reported a quantity-dependent effect for the risk of cancer recurrence as well, in their analysis of cancer patients undergoing colorectal surgery. The OR for recurrence risk was higher for ≥5 transfused units (OR, 2.02; 95% CI, 1.65-2.48, p<0.00001) as for 1-2 units (OR, 1.40; 95% CI, 1.18-1.67; p<0.0001) (16).

In contrast, we could not show a negative impact of RBC transfusion on the likelihood of responding to IT if RBC transfusions were administered within 30 days before the initiation of treatment. A study of Liu et al. (n=80 patients) demonstrated that the strongest increase (p<0.05) of immunosuppressive Tregs was apparent one day after RBC transfusion (11). Based on these findings, we assume that a negative influence on therapy response of IT requires a strong simultaneous interference with the immune system to mediate a worse outcome. Considering other immunomodulatory events, there was a trend of a higher irAE rate in the transfused group, although we would have expected a reduced rate. Additionally, a higher clinically significant infection rate before treatment start in the transfusion group was documented. We assume differentiating pathways between infection, irAE and therapy response to IT. Further evaluation of these special observations is not possible based on our data, thus meriting larger studies.

Although for ethical reasons, the effect of RBC transfusions can only be studied using such real-world data, nevertheless, several limitations have to be considered when interpreting our findings. First, our study population is small. More so, considering the applied inclusion criteria, a selected patient population was studied. Second, due to the broad use of IT in different cancer entities, the study population is heterogeneous. However, we primarily assume an immunomodulatory effect of RBC transfusion which is independent of the tumor entity. A statistical limitation is the expectable small percentage of transfused patients of the total study population. Indeed, a prospective study on transfusion needs in cancer care showed that 14.9% of patients were treated with a blood transfusion (18). Hence, larger prospective case-control studies are needed to confirm our findings and to improve the understanding of the specific underlying pathomechanism.

In conclusion, we demonstrate that the concomitant administration of RBC concentrates negatively influences the response rate to IT. We hypothesize that this effect is most likely mediated by immunosuppression exerted by RBC concentrates and independent from age, sex, ECOG, concomitant therapy and LDH. The results of our study do not justify the omission of a necessary transfusion; however, our findings might implicate a more restrictive transfusion management in patients undergoing IT and encourage the use of alternatives, such as erythropoietin to treat tumor-induced or treatment-associated anemia, if feasible.

## Data availability statement

The raw data supporting the conclusions of this article will be made available by the authors, without undue reservation.

## Ethics statement

The studies involving human participants were reviewed and approved by ethics committee of the Medical Department of the University Bonn. Written informed consent for participation was not required for this study in accordance with the national legislation and the institutional requirements.

## Author contributions

RM and STH organized the database, performed the statistical analysis and wrote the first draft of the manuscript. RM, STH, and AH contributed to conception and design of the study. AH conceived the original idea. AH and PB supervised the project. All authors contributed to manuscript revision, read, and approved the submitted version.

## Acknowledgments

We thank Guido Luechters for his statistical support, Stefanie Andrea Erika Held, Bettina Enste and Jessica von Fischer-Treuenfeld for their help to complete the dataset as well as Chrystel Flores for her support designing the figures.

## Conflict of interest

The authors declare that the research was conducted in the absence of any commercial or financial relationships that could be construed as a potential conflict of interest.

## Publisher’s note

All claims expressed in this article are solely those of the authors and do not necessarily represent those of their affiliated organizations, or those of the publisher, the editors and the reviewers. Any product that may be evaluated in this article, or claim that may be made by its manufacturer, is not guaranteed or endorsed by the publisher.

## References

[1] Steven A Fisher SA Robinson BW . Immunotherapy for lung cancer. Respirology 21(5):821–33. doi: 10.1111/resp.12789 27101251

[2] Kumar P Saini S Prabhakar BS . Cancer immunotherapy with check point inhibitor can cause autoimmune adverse events due to loss of treg homeostasis. Semin Cancer Biol 64:29–35. doi: 10.1016/j.semcancer.2019.01.006 30716481

[3] Meriggi F Zaniboni A . Antibiotics and steroids, the double enemies of anticancer immunotherapy: A review of the literature. Cancer Immunol Immunother (2021) 70(6):1511–17. doi: 10.1007/s00262-020-02786-3 PMC10991597 33165628

[4] Cortellini A Di Maio M Nigro O Leonetti A Cortinovis DL Aerts JG . Differential influence of antibiotic therapy and other medications on oncological outcomes of patients with non-small cell lung cancer treated with first-line pembrolizumab versus cytotoxic chemotherapy. J Immunother Cancer 9(4):e002421. doi: 10.1136/jitc-2021-002421 PMC8031700 33827906

[5] Hussain N Naeem M Pinato DJ . Concomitant medications and immune checkpoint inhibitor therapy for cancer: Causation or association? Hum Vaccin Immunother (2021) 17(1):55–61. doi: 10.1080/21645515.2020.1769398 32574106 PMC7872020

[6] Ricciuti B Dahlberg SE Adeni A Sholl LM Nishino M Awad MM . Immune checkpoint inhibitor outcomes for patients with non-Small-Cell lung cancer receiving baseline corticosteroids for palliative versus nonpalliative indications. J Clin Oncol (2019) 37:1927–34. doi: 10.1200/JCO.19.00189 31206316

[7] Iqbal N Haider K Sundaram V Radosevic J Burnouf T Seghatchian J . Red blood cell transfusion and outcome in cancer. Transfus Apher Sci (2017) 56:287–90. doi: 10.1016/j.transci.2017.05.014 28602484

[8] Kormi SM Seghatchian J . Taming the immune system through transfusion in oncology patients. Transfus Apher Sci (2017) 56:310–6. doi: 10.1016/j.transci.2017.05.017 28651910

[9] Petrelli F Ghidini M Ghidini A Sgroi G Vavassori I Petrò D . Red blood cell transfusions and the survival in patients with cancer undergoing curative surgery: A systematic review and meta-analysis. Surg Today (2021) 51:1535–57. doi: 10.1007/s00595-020-02192-3 33389174

[10] Almizraq RJ Seghatchian J Acker JP . Extracellular vesicles in transfusion-related immunomodulation and the role of blood component manufacturing. Transfus Apher Sci (2016) 55(3):281–91. doi: 10.1016/j.transci.2016.10.018 27865649

[11] Liu Y Sun J Xia Y Lyake MR Yu J . Effect of intraoperative blood transfusion on treg and FOXP3 in patients with digestive tract malignancies and different ABO blood types. BMC Anesthesiol (2021) 21:110. doi: 10.1186/s12871-021-01330-9 33838641 PMC8035765

[12] Long K Meier C Bernard A Williams D Davenport D . Woodward J. T-cell suppression by red blood cells is dependent on intact cells and is a consequence of blood bank processing. Transfusion (2014) 54:1340–7. doi: 10.1111/trf.12472 PMC4344125 24188586

[13] Cata JP Wang H Gottumukkala V Reuben J Sessler DI . Inflammatory response, immunosuppression, and cancer recurrence after perioperative blood transfusions. Br J Anaesth (2013) 110:690–701. doi: 10.1093/bja/aet068 PMC3630286 23599512

[14] Darvin P Toor SM Sasidharan Nair V Elkord E . Immune checkpoint inhibitors: Recent progress and potential biomarkers. Exp Mol Med (2018) 50:1–11. doi: 10.1038/s12276-018-0191-1 PMC6292890 30546008

[15] Abdel-Razeq H Hashem H . Recent update in the pathogenesis and treatment of chemotherapy and cancer induced anemia. Crit Rev Oncol Hematol (2020) 145:102837. doi: 10.1016/j.critrevonc.2019.102837 31830663

[16] Amato A Pescatori M . Perioperative blood transfusions for the recurrence of colorectal cancer. Cochrane Database Syst Rev (2006) CD005033. doi: 10.1002/14651858.CD005033.pub2 16437512 PMC6486137

[17] Aldea M Orillard E Mansi L Marabelle A Scotte F Lambotte O . How to manage patients with corticosteroids in oncology in the era of immunotherapy? Eur J Cancer 141:239–51. doi: 10.1016/j.ejca.2020.09.032 33212339

[18] Ludwig H van Belle S Barrett-Lee P Birgegård G Bokemeyer C Gascón P . The European cancer anaemia survey (ECAS): A large, multinational, prospective survey defining the prevalence, incidence, and treatment of anaemia in cancer patients. Eur J Cancer (2004) 40:2293–306. doi: 10.1016/j.ejca.2004.06.019 15454256

