# Supplementary material for: Red blood cell transfusions impact response rates to immunotherapy in patients with solid malignant tumors
Source: Front Immunol. 2022 Sep 8;13:976011. doi: 10.3389/fimmu.2022.976011 (PMC9492841; doi:10.3389/fimmu.2022.976011)
Supplement: Supplementary file 1 [file DataSheet_1.pdf]

Supplementary material

Supplementary Table

Supplementary Table 1 Patient characteristics of transfused and non-transfused patients (30 days before IT start)

	**total** (N = 55), n (%)	**RBC transfusion** (N = 9), n (%)	**no RBC transfusion** (N = 46), n (%)	p-value[a]
**age (years)**				
mean	62	55	64	0.094*
range	34-90	35-76	34-90	
elderly (>70)	15 (27.3)	2 (22.2)	13 (28.3)	1.000
**gender**				1.000
male	39 (70.9)	7 (77.8)	32 (69.6)	
female	16 (29.1)	2 (22.2)	14 (30.4)	
**ECOG**				1.000
0-1	35 (63.6)	6 (66.7)	29 (63.0)	
≥2	17 (30.9)	3 (33.3)	14 (30.4)	
**primarius**				0.421
lung	19 (34.5)	3 (33.3)	16 (34.8)	
skin	7 (12.7)	0 (0)	7 (15.2)	
head neck	10 (18.2)	3 (33.3)	7 (15.2)	
urogenital	5 (9.1)	0 (0)	5 (10.9)	
CUP	4 (7.3)	0 (0)	4 (8.7)	
breast	3 (5.5)	1 (11.1)	2 (4.3)	
others	7 (12.7)	2 (22.2)	5 (10.9)	
**one drug immunotherapy**				1.000
Atezolizumab	8 (14.5)	3 (33.3)	5 (10.9)	
Pembrolizumab/Nivolumab	34 (61.8)	4 (44.4)	30 (65.2)	
Ipilimumab	1 (1.8)	0 (0)	1 (2.2)	
**two drug immunotherapy**				
Nivolumab+Ipilimumab	12 (21.8)	2 (22.2)	10 (21.7)	
**additional therapy**				
additional chemotherapy	15 (27.3)	4 (44.4)	11 (23.9)	0.236
additional radiotherapy	11 (20.0)	3 (33.3)	8 (17.4)	0.362
**line of therapy**				0.166
1	14 (25.5)	3 (33.3)	11 (23.9)	
2	15 (27.3)	0 (0)	15 (32.6)	
3	13 (23.6)	4 (44.4)	9 (19.6)	
4	5 (9.1)	1 (11.1)	4 (8.7)	
≥5	8 (14.5)	1 (11.1)	7 (15.2)	
mean	2.7	2.7	2.7	
**number of organs with metastasis**				0.070*
mean	1.7	2.3	1.7	
**LDH**				0.051*
mean	317	704	238	

[a]was calculated using Fisher's exact/*Wilcoxon

RBC = red blood cell, CUP = cancer of unknown primary
